# Neonatal adiposity may increase plasmatic cytokines

**DOI:** 10.1371/journal.pone.0238370

**Published:** 2020-09-04

**Authors:** Maria Hernandez-Trejo, Reyna Sámano, Gabriela Chico-Barba, Maria Luisa Pizano-Zarate, Norma Estela Herrera-González

**Affiliations:** 1 Neurobiología del Desarrollo, Instituto Nacional de Perinatología, Secretaría de Salud, Ciudad de México, México; 2 Nutrición y Bioprogramación, Instituto Nacional de Perinatología, Secretaría de Salud, Ciudad de México, México; 3 Sección de Posgrado e Investigación, Escuela Superior de Medicina del Instituto Politécnico Nacional, Plan de San Luis y Díaz Mirón s/n, Casco de Santo Tomás, Ciudad de México, México; University of Mississippi Medical Center, UNITED STATES

## Abstract

Maternal health and nutritional status before and during gestation may affect neonates' immune system and energy balance as they develop. The objective of this study was to associate certain clinical markers of maternal adiposity (body mass index and gestational weight gain) and neonatal adiposity (birth weight, abdominal circumference, and waist/height index) with the levels of pro- and anti-inflammatory cytokines in umbilical cord blood at birth: IL-1β, IL-1Rα, IL-4, IL-6, IL-10, IFN-γ, and TNF-α. An exploratory cross-sectional study was conducted with a convenience sample of women from one hospital recruited shortly before giving birth through scheduled cesarean section. Of 31 the pregnant women who agreed to participate and met the inclusion criteria, twenty-nine newborns from these women were analyzed. Three cases of tobacco smoking during pregnancy were identified as an unexpected maternal risk factor and were included in the analysis. Typical of the population treated at this hospital, ten of our participants had diabetes during pregnancy, and nine of them had a pre-pregnancy BMI> 25. Non-parametric statistical analyses and a generalized linear model with gamma scale response with a log link were performed. Results: Correlation analyses, differences in medians, and a prediction model all showed positive and significant results between cytokine levels in cord blood and neonatal abdominal circumference, birth weight, and waist-height index. For maternal variables, smoking during pregnancy showed significant associations with cytokine levels in cord blood. Conclusion: This study found a variety of associations suggesting that increased neonatal adiposity increases pro-inflammatory cytokine levels at birth.

## Introduction

Cytokines can act as hormones in the immune system and are generally influenced by nutritional status in the same way that other physiological systems would be [1].

Neonatal cytokines levels may be good indicators of future health status. There is a compelling rationale for measuring neonatal cytokines, as there is a paucity of investigation on using neonatal cytokine levels as indicators.

Maternal nutritional status is one of the most dominant contributors to a fetal environment that potentially modifies a newborn's physiology [2–4]. Obesity is associated with a meta-inflammatory state characterized by increased cytokines from adipose tissue [5]. Obese mothers tend to have increased levels of pro-inflammatory (IL-6, TNF-α, IL-8, IL-1β) and counter-regulatory (IL-1Rα) cytokines in maternal serum than healthy weight women [6–8].

Very little, if any, is known about the impact that fetal overnutrition (expressed as high birth weight and excess neonatal adiposity) has on the immune system of newborns. Ninety percent of body fat at birth is deposited in the last ten weeks of gestation, and maternal concentrations of circulating free fatty acids regulate the provision of lipids to the fetus [9, 10]. Free fatty acids (FFAs) are transferred to the fetus to a greater extent through the placenta during pregnancies complicated by obesity or diabetes (even with the normal range of glycemia), explaining why these morbidities are associated with increased fat mass in newborns. Moreover, FFAs are associated with insulin resistance and inflammation that increase the transfer of lipids through the placenta earlier in gestation [11–14].

Neonates with higher fat mass could have prenatal increased exposure to FFAs [14, 15]. Fatty acids released by adipocytes can produce a robust pro-inflammatory signal by binding to toll-like receptors on the surface of local macrophages and inducing the pro-inflammatory transcription factor NF-kβ [16, 17].

This article reports the analysis of an exploratory study conducted with twenty-nine mother/child binomials on the associations between pre-pregnancy body mass index (P-BMI), gestational weight gain GWG), neonatal abdominal circumference (AC), waist/height index, and weight at birth on the levels of seven cytokines in the umbilical cord blood at birth. As secondary results, information on another risk factor—exposure to tobacco smoking—was also gathered and associated with the same cytokines.

## Methods

In this exploratory cross-sectional study, we examined possible associations between neonatal cytokines and maternal and neonatal adiposity clinical markers that may influence fetal development. This project was carried out between February and June of 2017 at the Instituto Nacional de Perinatologia in Mexico City after being approved by the relevant Investigation Committee, Research Ethics Committee, and Biosafety Committee (registration number 384-212250-22651).

Inclusion criteria were: being a pregnant woman over the age of 18 years, carrying a singleton pregnancy, being scheduled for an elective cesarean section, and not yet having gone into labor. Only women with a scheduled cesarean section and who had not yet gone into labor were included in the study to control for the inflammatory influence of labor and vaginal delivery. Exclusion criteria included: the use of any antimicrobials or steroids in the previous four weeks or a diagnosis of any health condition known to influence maternal/neonatal cytokine levels (labor, hypertension, preeclampsia, autoimmune disease, congenital malformations, premature rupture of membranes, or active infection).

**Fig 1:**
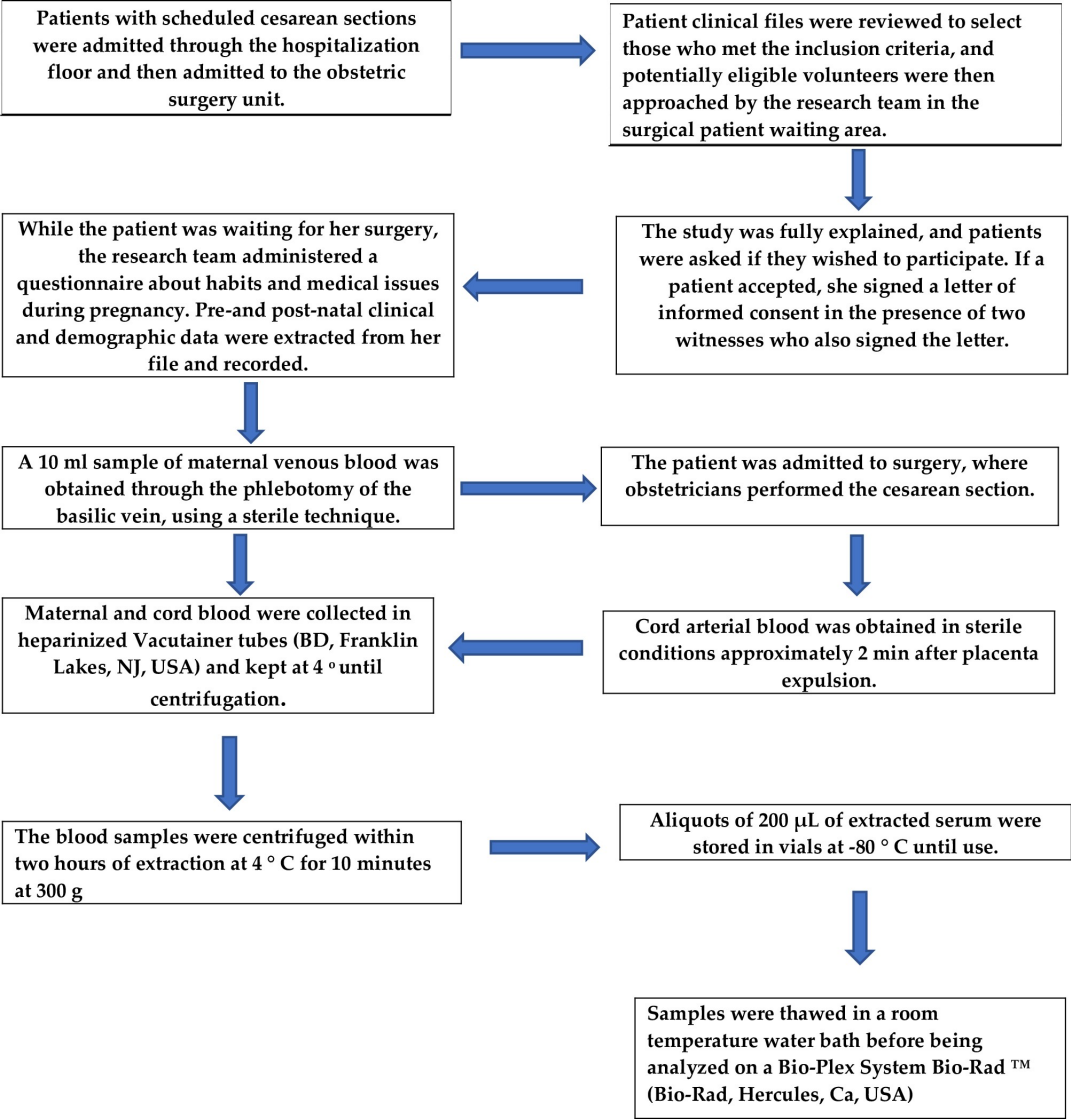
Data collection and sample processing.

A convenience sample of eligible patients was recruited in a consecutive fashion in the hospital's labor and delivery room shortly before giving birth by scheduled cesarean section. There, the study was explained to them, and they signed informed consent—in the presence of two witnesses who also signed the same document—for the collection of clinical data and biological material. Maternal and neonatal clinical/sociodemographic data, obstetric outcomes, and behavioral habits were retrieved from medical records and recorded (Fig 1).

The study initially commenced with 31 participants, but two were excluded because their maternal venous blood sample was obtained after a cesarean section. Taking a sample after a cesarean—because of surgical manipulation and the administration of drugs and/or anesthetics—is known to modify the parameters of the cytokines in newborns.

### Nutritional status assessment

All participants were asked to report their pre-pregnancy weight and height. Self-reported pre-pregnancy weight and height are useful and reliable proxies due to their adequate correlation with real measurements [18]. With this information, we calculated participants' pre-pregnancy BMI using World Health Organization guidelines and expressed it as kg/m^2^ [19]. Women's anthropometric measurements were taken by trained nurses using the Lohman technique [20]. Gestational weight gain was obtained by subtracting the pre-gestational weight from the final gestational weight. Final gestational weight was measured using a digital scale (TANITA model BWB-800, with an accuracy of 100 g) at some point between one week before and up until 12 hours before childbirth.

The Mexican population has a high prevalence of obesity and overweight (40% and 36%, respectively), and an estimated diabetes mellitus prevalence of 11% [21]; gestational diabetes is calculated between 8.7 and 17.7% [22]. Patients with these conditions make up a significant proportion of the population seeking care at the institution where the study was conducted, which is why it is not surprising that six cases in our study had gestational diabetes, and four had pre-existing diabetes mellitus. We combined these two situations in a single group, entitled "Diabetes during pregnancy."

Neonatal weight (g), length (cm), and abdominal, thoracic, and head circumferences (cm) were obtained at birth by trained pediatricians using the Lohman technique [20]. A SECA-brand 354-model scale (accuracy 0.010 kg) was used to measure neonatal weight; a SECA-brand 203-model infantometer (accuracy 0.10 cm) was used for the length; circumferences were measured with a SECA-brand 201-model tape measure (accuracy 0.10 cm). The infant waist/height index was obtained by the quotient of the abdominal circumference in centimeters and the height in centimeters of the newborns in the first minutes after birth. The neonatal weight classification by gestational age was established in accordance with the relevant Official Mexican Standard [23].

### Blood samples

Fig 1 shows how patients were recruited and enrolled in the study, their data, and how samples were processed.

### Measurement of cytokines

Maternal blood and neonatal arterial cord blood using a Bio-Plex Pro Human 27-plex assay according to the manufacturer's instructions (#M500KCAF0Y, BioRad, Hercules, CA, USA). Samples were processed in a Bio-Plex System, BioRad.

We selected seven cytokines (IL-1β, IL-1Rα, IL-4, IL-6, IL-10, IFN-γ, and TNF-α) to be representative of pro-inflammatory and anti-inflammatory cytokine groups.

### Statistical analyses

Results were analyzed using the IBM SPSS Statistics for Windows, Version 23.0 (Armonk, NY: IBM Corp). Data in tables are presented as median and interquartile range. Correlations between neonatal cytokines and maternal and newborn nutritional status were assessed using the Spearman correlation coefficient. Differences in somatometry at birth between the P-BMI and maternal diabetes groups were evaluated with the Kruskal-Wallis test. Differences in neonatal cytokines levels between groups were compared with a Mann-Whitney U. The CRT (classification decision trees) method of the CART model was used to identify the cut-off points of the maternal and neonatal variables that showed significance in the Mann-Whitney U analysis. Significant results were defined as those with a probability of α-error < 0.05.

The gamma scale response with log link regression of the Generalized Linear Model was performed to assess the independent effect of maternal clinical adiposity measures (P-BMI and GWG), and clinical adiposity measures (abdominal circumference, weight at birth, and waist/length index) on umbilical cord blood cytokines levels.

### Sampling power calculation

Due to the small sample size, a post hoc power calculation was carried out after data collection, using the G*Power analytical program. A β value was calculated with an exact post-hoc test for bivariate correlation for non-parametric differences between groups.

## Results

All women were Mexican, from a middle or middle/low socioeconomic background, and had no access to other formal healthcare service services. Table 1 shows the clinical nutrition features of the 29 mothers and their offspring. The patient sample had a median pre-pregnancy BMI of 25.5, with a median gestational weight gain of 11.0 kg.

**Table 1:** General clinical characteristics of mothers and children. N = 29.

	Median	IQR	Min–Max
**Mother**			
**Maternal age** (years)	31	26.5–38.5	19–43
**Pregestational weight** (Kg)	61	57–71.5	50–103
**Pre-pregnancy BMI** (Kg/m^2^)	25.5	23.3–28.5	20.9–44.5
**Gestational weight gain** (Kg)	11.0	6.95–15.5	-4.2–22
**Number of pregnancies**	3	2–4	1–5
**Number of deliveries**	0.0	0–0.5	0–2
**Number of abortions**	0.0	0–1.0	0–1
**Number of previous cesarean sections**	1.0	1.0–2.0	0–3
**Newborn**			
**Gestational age at birth (weeks)**	38.4	37.6–38.8	37.1–41
**Weight at birth (g)**	2970	2822–3250	2510–3765
**Height at birth** (cm)	48.0	47.0–50.0	45–52
**Neonatal head circumference** (cm)	34.5	34.5–35.2	32.5–37.0
**Neonatal thoracic circumference** (cm)	32.0	31.2 - -33.0	29.0–34.0
**Neonatal abdominal circumference** (cm)	30.0	29.0–31.7	28.0–33.5
**Waist-height index**	0.622	0.601–0.641	0.57–0.69

BMI: Body Mass Index.

SD: Standard deviation.

IQR: Interquartile Range.

Min—Max: Minimum and maximum values.

### Characteristics of the mothers

Nine women with diabetes (pre-existing and gestational) had overweight or obesity and one normal-weight woman had pre-existing diabetes mellitus. As these comorbidities are endemic and uncontrolled in our population, it places us in the position of studying the effect that these morbidities can have on newborns. Three pregnant women smoked during gestation (one of them had gestational diabetes).

### Characteristics of the newborns

All neonates presented an Apgar score of 8 and 9 at 1:00 and 5:00 minutes, respectively, and were born healthy (pediatrician's diagnosis). Fifteen newborns were girls, and fourteen were boys. The neonates were born with an adequate weight for their gestational age.

There were no statistically significant differences between children born to mothers with normal, overweight, and obese P-BMIs in any of their anthropometric values, including birth weight (p = 0.7), length (p = 0.9), waist/height index (p = 0.8), and abdominal circumferences (p = 0.9). Similarly, no significant difference was found when comparing children born to a diabetic mother with P-BMI ≥ 25 and diabetes (n = 9), against those born to a non-diabetic mother with a P-BMI ≥ 25 (n = 8), to a non-diabetic mother with P-BMI <25 (n = 11), or to a diabetic mother with P-BMI<25 (n = 1): weight (p = 0.5), length (p = 0.9), waist/height index (p = 0.3), and abdominal circumferences (p = 0.4).

### Spearman's correlation of maternal and neonatal nutritional status with cord blood cytokines levels

Table 2 shows positive and significant correlations between plasma cytokine levels at birth and variables clinically related to neonatal growth and adiposity. For instance, IL-1β correlated with all neonatal variables; IL-1Rα correlated with abdominal circumference at birth and waist/height index. Of the maternal variables: age was positively correlated with IFN-γ and GWG with IL-1β. Statistical power was moderate to high.

**Table 2:** Spearman correlation coefficient for neonatal cytokines, by maternal and newborn nutritional status. rho / p-value. (n = 29).

	Maternal age (years)		Pre-pregnancy BMI (m/kg^2^)	Gestational weight gain (kg)		Birth weight (g)		Length at birth (cm)		Abdominal circumference at birth (cm)		Waist/Height Index	
**IL-1β**	0.20/0.29		-0.30/0.10	**0.43/0.01**	**	**0.47/0.01**	**	**0.37/0.04**	*	**0.67/0.001**	§	**0.39/0.03**	*
**IL-1Rα**	0.12/0.50		-0.23/0.22	0.28/0.13		0.15/0.43		-0.05/0.76		**0.45/0.01**	**	**0.50/0.001**	§
**IL-4**	0.23/0.21		-0.31/0.09	0.24/0.20		**0.40/0.02**	**	0.23/0.21		**0.51/0.001**	§	0.32/0.08	
**IL-6**	0.1/0.40		-0.21/0.27	0.26/0.17		0.30/0.11		0.21/0.27		**0.61/0.001**	§	**0.44/0.01**	**
**IL-10**	0.01/0.95		-0.10/0.59	0.02/0.90		-0.12/0.51		-0.07/0.71		0.26/0.15		0.325/0.08	
**IFN-γ**	**0.39/0.039**	*	-0.12/0.53	0.11/0.54		0.26/0.17		0.23/0.22		**0.52/0.001**	§	0.28/0.14	
**TNF-α**	0.24/0.27		-0.02/0.89	-0.09/0.68		0.16/0.47		-0.09/0.66		0.26/0.23		0.29/0.18	

p = significance (α value ≤ 0.05) in bold.

* Statistical power >0.5

** Statistical power ≥0.70

**§** Statistical power ≥ 85

BMI: Body mass index

### Differences of medians between groups

Table 3 shows that infants with greater abdominal circumference, waist/height ratio, and birth weight all had significantly increased levels of plasma pro-inflammatory cytokines, all with moderate and high statistical powers for the sample size of 29 cases. None of the maternal variables were significantly different between groups.

**Table 3:** Umbilical cord blood cytokines (pg/mL) by clinical characteristics. Mann-Whitney U (significance). n = 29.

	IL-1β	IL-1Rα	IL-4	IL-6	IL-10	IFN-γ	TNF-α
**Birth Weight** ^**a**^ **(g) ≥ 2877 (20) *vs*. < 2877 (9)**	19.5**	50.5	**23.0**	45.0*	73.0	36.0*	18.0
	**(0.001)**	(0.06)	**(0.002)**	**(0.03)**	(0.4)	**(0.02)**	(0.5)
**Neonatal Waist/height Index** ^**a**^ **≥ 0.629 (13) *vs*. < 0.629 (16)**	54.0*	39.0*	70.5	51.0*	61.0	66.0	37.0
	**(0.028)**	**(0.004)**	(0.14)	**(0.02)**	(0.59)	(0.14)	(0.12)
**Neonatal abdominal Circumference** ^**a**^ **(cm) ≥29.5 (19) *vs*. <29.5 (10)**	18.5**	40.5*	31.0**	28.0**	63.0	28.0**	36.0
	**(0.000)**	**(0.01)**	**(0.002)**	**(0.001)**	(0.1)	**(0.004)**	(0.40)
						
**Pre-pregnancy BMI** ^**a**^ **≥25 (17) *vs*. <25 (12)**	90.0	94.0	89.0	98.0	100.5	95.0	54.0
	(0.61)	(0.74)	(0.58)	(0.85)	(0.94)	(0.98)	(0.72)
**GWG** ^**a**^ **(Kg) ≥10 (19) *vs*. <10 (10)**	60.0	61.0	93.5	77.0	83.0	83.0	34.0
	(0.11)	(0.12)	(0.94)	(0.42)	(0.60)	(0.92)	(0.21)
						
**Diabetes during gestation Yes (10) *vs*. No (19)**	88.5	80.0	94.5	93.0	88.0	68.0	45.0
	(0.76)	(0.51)	(0.98)	(0.94)	(0.76)	(0.41)	(0.48)
						
**Smoking during pregnancy Yes (3) *vs*. No (26)**	15.0*	12.0**	27.0	17.0*	22.5	23.0	8.0
	**(0.09)**	**(0.05)**	(0.42)	(0.13)	(0.25)	(0.31)	(0.21)
**Sex at birth Female (15) *vs*. Males (14)**	0.17 (0.3)	22.1 (0.4)	0.81 (0.1)	0.22 (0.7)	0.31 (0.1)	13.7 (0.4)	4.89 (0.2)

p <0.05, in bold.

^**a**^ Cut off points from CART model (CRT method)

* Statistical power >0.5

** Statistical power ≥ 0.70

GWG: Gestational weight gain

BMI: Body mass index

### Predictive model

Table 4 explains the ability of the independent variables to predict cytokine levels in umbilical cord blood with statistical significance. The model showed that smoking during pregnancy can positively and significantly predict levels of IL-1β, IL-1-Rα, IFN-γ, and TNF-α in cord blood. The results on P-BMI and GWG showed negative predictions. On the other hand, neonatal abdominal circumference positively predicted the concentration of IL-1β, IL-6, IL-10, IL-4, and IFN-γ in neonatal plasma.

**Table 4:** Clinical predictors of neonatal cytokines plasmatic levels at birth (pg/mL)^♦^. Generalized Linear Model (gamma with log link). n = 29.

	B	CI 95% (Wald)	Sig*
**IL-1β**			
**AC**	0.228	0.125, 0.332	<0.001
**Pre-BMI**	-0.041	-0.055, -0.028	<0.001
**GWG**	-0.023	-0.042, -0.003	0.021
**Smoking**	0.680	0.198, 1.163	0.006
**IL-1Rα**			
**Pre-BMI**	-3.443	-6.435, -0.451	0.024
**Smoking**	110.02	8.159, 211.8	0.034
**IL-6**			
**AC**	0.936	0.449, 1.423	<0.001
**Pre-BMI**	-0.215	-0.287, -0.145	<0.001
**GWG**	-0.135	-0.237, -0.032	0.01
**IFN-γ**			
**AC**	27.066	13.193, 40.939	<0.001
**Pre-BMI**	-5.432	-7.504, -2.359	<0.001
**GWG**	-5.11	-7.864, -2.359	<0.001
**Smoking**	58.538	16.372, 100.70	0.026
**IL-10**			
**AC**	0.316	0.127, 0.505	0.001
**Pre-BMI**	-0.072	-0.118, -0.026	0.002
**GWG**	-0.058	-0.108, -0.008	0.023
**IL-4**			
**AC**	0.743	0.255, 1.231	0.003
**Pre-BMI**	-0.237	-0.322, -0.153	<0.001
**GWG**	-0.156	-0.255, -0.056	0.002
**TNF-α**			
**Pre-BMI**	-1.142	-2.067, -0.217	0.016
**GWG**	-1.210	-0.909, -0.512	0.001
**Smoking**	8.450	0.130, 16.769	0.047

^♦^ Only results with statistical significance are presented

* Wald's square chi

AC: Abdominal circumference at birth

Pre-BMI: Pregestational body mass index

GWG: Gestational weight gain

## Discussion

The original intention of this exploratory study was to establish the convenience of doing a large comparative study. In this sense, the results obtained were encouraging, as they were statistically significant and supported by moderate and high statistical powers.

### Maternal pregestational-BMI and gestational weight gain

The influence of the mother's P-BMI and GWG on the pro-inflammatory state of her infant at birth had been previously studied, notably by Dosch et al., who found higher values of PCR and TNF-α in cord blood in pregnancies complicated by obesity [24].

Like us, Vega and Estampador [25, 26] reported negative correlations of IL-6 with P-BMI. However, our results were not statistically significant and might only be conclusive with a larger sample size.

### Neonatal clinical adiposity

For newborns, it has been documented that being born to an overweight or obese mother [27]or one with excessive gestational weight gain is associated with a significant increase in adipose tissue [28, 29]. Moreover, maternal glycemia is an important contributor to neonatal adiposity, independent of pregestational BMI [13].

Abdominal circumference, birth weight, and waist/height index are good clinical markers of adiposity in newborns [30–32]. Abdominal circumference is also a predictor of risk of chronic noncommunicable diseases in the short- and medium-term and is additionally related to school-age adiposity [33]. In our neonates, AC was moderately correlated with an increase in their mothers' gestational weight (r = 0.45, p = 0.016), but not with P-BMI.

Current evidence suggests that maternal lipids (free fatty acids and their source, triglycerides) from obese, glucose-tolerant women are contributing to excess fetal fat deposition starting from the first half of pregnancy [15, 34]. In addition, maternal glycemia also contributes to increased neonatal adiposity, seemingly independent of the pre-pregnancy body mass index [13, 34, 35]. McCloskey reported that neonatal AC and the waist/height index had a moderate positive correlation with both pro-inflammatory cytokines (IL-1β, IL-6, and IFN-γ), as well as with IL-1Rα and IL-4 [36]. Alike, in our newborns, when the three markers of neonatal adiposity—birth weight, waist/height ratio, and AC—were higher, median neonatal plasmatic cytokine concentrations were also higher. Few reports on the levels of cytokines at birth exist in international literature. Aye and Pantham [8, 9], postulate that no pro-inflammatory activation occurs in human fetuses of obese mothers, while other authors believe that fetal inflammation can occur in cases of severe obesity (grades II and III) [24].

The generalized linear model confirmed a pro-inflammatory predictive pattern in umbilical cord blood associated with the abdominal circumference at birth. These results lead us to posit that AC at birth, and other markers of neonatal adiposity, influences the immunological state at birth, probably more than maternal fat mass. Other authors have also found an association between increased fetal growth and inflammatory cytokines, such as IL-6 and TNF-α [37]. An interesting study carried out in 2006 in pregnant adolescents found results very similar to ours: inflammatory cytokines IL-6 and TNF-α in umbilical cord were significantly associated with increased fetal growth [38].

The inflammatory state we observed in this sample of newborns may not have been exclusively mother-induced, and it is entirely possible that it was produced by an immune response from the newborn's own increased adipose tissue. This is certainly an area to further speculate on and to study. Numerous statistical and epidemiological observations show direct correlations between adult obesity with childhood and neonatal obesity, and the association between the distribution of body fat in young children and metabolic and cardiovascular risk later in life [39–41].

### Smoking during pregnancy

An unexpected finding in this study was the three cases of smoking during pregnancy; this small sample provided significant associations in the statistical analysis. We observed that the three cases of maternal tobacco use during pregnancy were able to predict the elevation of levels of the neonatal cytokines IL-1β, IL-1R α, IFN-γ, and TNF-α. Infants of smokers also had higher levels of cytokines IL-1β, IL-1R α. In contrast with our results, Almanzar found no differences in cytokine levels among the newborns of smoking mothers versus non-smoking mothers [42]. This discussion is important because maternal smoking can be a fetal programming factor that induces inflammation. Maternal tobacco use during pregnancy is recognized to increase a child's lifetime risk of repetitive respiratory infections, bronchitis, and asthma [43]. Fetal exposure to tobacco combustion products is considered passive smoking and is associated with an increased risk of obesity and metabolic syndrome in children [44, 45]. To understand the mechanisms of cytokine elevation that occur due to smoking, Chen proposes that the most common situation is the activation of transcription factors [46].

### Diabetes during pregnancy

In this study, there were no differences in cytokine levels between infants of mothers with diabetes and infants of mothers without diabetes. This is undoubtedly an important area of research that requires a larger sample size to draw conclusions.

## Conclusions

Clinical variables related to fat mass in neonates were associated with higher levels of pro-inflammatory cytokines at birth, with abdominal circumference being the most significant outcome. Our study was unable to conclusively establish associations between maternal clinical fat and pro- and anti-inflammatory cytokine levels in cord blood. More studies are needed to benchmark average values of cytokines at birth and the changes that cytokines undergo due to environmental and maternal health conditions as part of the fetal programming process.

## Strengths and limitations

Due to the salient ethical considerations of studying human newborns, neonatal cytokine levels is a little-studied topic, and we performed this pilot study as we could not find reliable reference values to calculate the variability of cytokines as birth. The initial intention of this project was to evaluate the convenience of performing a larger comparative study. In this sense, our results are encouraging, as our principal results were statistically significant and supported with moderate and high statistical powers. We hope that this study may be an approach to understanding how neonatal adiposity can be associated with newborns' cytokines and that these can be an area of future research.
